# Physical Properties, Cytocompatibility and Sealability of HealApex (a Novel Premixed Biosealer)

**DOI:** 10.22037/iej.v13i3.20188

**Published:** 2018

**Authors:** Zahra Shourgashti, Hamid Keshvari, Hasan Torabzadeh, Mostafa Rostami, Shahin Bonakdar, Saeed Asgary

**Affiliations:** a *Faculty of Biomedical Engineering, Amir-Kabir University of Technology, Tehran, Iran; *; b *Dental Research Center, Research Institute of Dental Sciences, Dental School, Shahid Beheshti University of Medical Sciences, Tehran, Iran; *; c *National Cell Bank, Pasteur Institute of Iran, Tehran, Iran;*; d *Iranian Center for Endodontic Research, Research Institute of Dental Sciences, Shahid Beheshti University of Medical Sciences, Tehran, Iran*

**Keywords:** Biocompatibility, Calcium Silicate Phosphate, Calcium Enriched Mixture, CEM Cement, Dental Leakage, Endodontic, HealApex, Physical Properties, Sealer

## Abstract

**Introduction:**

The objective of this study was to evaluate the physical properties, cytotoxicity and sealing ability of HealApex _a new premixed calcium-silicate-phosphate-based biosealer_ in comparison with AH-26.

**Methods and Materials:**

Setting time, working time, film thickness, flow and radiopacity evaluation were performed according to ISO 6876 specification. L929 fibroblasts were incubated with the extracts of sealers and cytotoxicity was then evaluated using MTT assay. Thirty intact extracted human premolars were instrumented using step-back technique. The specimens were obturated with gutta-percha and experimental sealers employing lateral condensation technique. Sealing ability of sealers was investigated for up to one month using fluid filtration method. Data were statistically analyzed by *t*-test and ANOVA.

**Results:**

Physical properties of both sealers conformed to ISO specification. AH-26 exhibited significantly higher flow, higher radiopacity and lower film thickness; whereas HealApex showed lower setting time (*P*<0.05). HealApex represented high cell viability (*P*<0.05); however, AH-26 demonstrated significantly lower cell viability compared with the negative control group (*P*<0.05). There was no significant difference in microleakage between the sealers after 1 and 7 days; however, after 30 days, HealApex displayed better sealing ability (*P*<0.05).

**Conclusions:**

In this *in vitro* study, HealApex revealed acceptable physical properties, biocompatibility and good sealing ability as an endodontic sealer. Obtained results showed the new sealer had acceptable physical properties and good biocompatibility. In short term, the sealing ability of HealApex was comparable with AH-26 whilst in long term, HealApex’s sealing ability was better than the epoxy resin-based sealer.

## Introduction

The main objective of root canal obturation is to provide a hermetic three-dimensional seal. As the core filling material itself does not bond to the dentin of root canal walls, the association of an endodontic sealer along with the core filling material is necessary to prevent apical microleakage [1]. 

An ideal root canal sealer should possess certain characteristics, including biocompatibility, sealing ability, dimensional stability, low solubility, good adhesion to dentin, slow setting time, radiopacity, antibacterial activity and the ability to allow or induce bone repair [2, 3]. New materials are being continuously launched into the market to meet all of these important properties. 

Some examples of sealers are based on zinc oxide eugenol, resin, silicone, glass ionomer, calcium hydroxide and calcium phosphate. Most of them have shown different levels of weakness in biocompatibility, dimensional stability, solubility, adherence to dentin, leakage, bond strength and handling properties [4].

Calcium silicate-based materials with promising clinical outcomes [5, 6] have recently been introduced as root canal sealers [7]. They are biocompatible, non-shrinking [8] and show alkaline pH during setting, which results in antibacterial activity [9]. These biomaterials were developed mainly because of their bioactivity. They release high amount of Ca^2+^ and OH^-^ ions, resulting in nucleation of calcium phosphates. They also induce characteristic markers mineralization process [10]. In contact with tissue fluids, they can also form hydroxyapatite. Such bioactivities can create a chemical bond between dentin and the sealing material [11]. Despite the above mentioned strengths, none of the commercial sealers of this category comply with all of the necessary requirements for the stability and adherence to dentin [12].

Recently, a new premixed calcium-silicate-phosphate-based biosealer has been developed (HealApex). The major components of this sealer are calcium silicates, calcium phosphate, and zirconium oxide, which are mixed with water-free polymeric vehicles. 

AH-26 (Dentsply DeTrey, Konstanz, Germany), a kind of epoxy resin-based material, has demonstrated acceptable handling and physical characteristics as well as excellent sealing properties it presents with significant toxicity specifically in the fresh state, which decreases in the set state [13, 14]. AH-26 is frequently used in endodontic studies to compare newly introduced root canal sealers.

The aim of the present study was to evaluate the physical properties, cytotoxicity and sealing ability of HealApex and compare them with those of AH-26.

## Materials and Methods

Sealers tested in this study are AH-26 silver free sealer (Dentsply DeTrey, Konstanz, Germany) and HealApex.

***Physical properties***

For each material, setting time, working time, film thickness and flow were measured three times according to International Organization for Standardization (ISO) 6876 (2012). The mean and standard deviation values were calculated and recorded.

***Radiopacity test***

Two acrylic sheets containing 5 wells, measuring 1 mm in thickness and 5 mm in diameter, were prepared. The sealers were poured into the wells and kept in an incubator at 37^º^C until the materials were completely set. Then, the thickness was checked with a digital caliper. Dentin specimens (1-mm thickness) were prepared by cutting freshly extracted human molar teeth with a diamond cutting disc (Mecatome, Presi, France). Thereafter, the specimens were placed on a GXS digital sensor along with an aluminum step wedge. Preliminary examination showed that the dentin and HealApex sealer had low radiopacity. Thus, for these samples, the high resolution step wedge, graduated from 0.5 to 5 mm Al (in 0.5-mm increments), was used. Highly radiopaque AH-26 was exposed along with an aluminum step wedge graduated from 1 to 10 mm Al (in 1-mm increments). 

Radiographs were taken using a dental x-ray machine (Gendex Intra-Oral x-ray**,** USA) operating at 65 kV and 7 mA, with exposure set at 0.32 sec and a focus-film distance of 30 cm. Digital images were imported to Digora for Windows software version 2.7 (Orion Corporation Soredex, Helsinki, Finland).The said software measures the density of radiographic images, at each step on the aluminum step-wedge, samples and specimens of dentin. An average of 5 readings for each specimen and aluminum step was recorded. These values were then converted into millimeters of aluminum in a manner similar to the method in Duarte *et al.* study [15].

***Cytotoxicity testing***

Cytotoxicity assessment was performed according to ISO 10993-5. L929 were obtained from National Cell Bank of Iran (NCBI), Pasteur Institute, Iran. The cells were grown in DMEM (Dulbecco modified Eagle medium) medium containing 10% fetal bovine serum (FBS), 100 U/mL penicillin and 100 µg/mL streptomycin in an atmosphere of 5% CO_2_ at 37^°^C in a humidified incubator. 

The cytotoxicity of materials was tested in 2 states - fresh and set. For the fresh states, a disc was placed at the bottom of 24-well plates. To prepare the set specimens, sealers were poured in cylindrical Teflon molds of 4 mm in diameter and 6 mm in height and then incubated to set. Three specimens were prepared for each material at each state. Before extraction, freshly mixed and set sealers were exposed to ultraviolet (UV) light for 2 h. The surface area to volume ratio used in the extract preparation was considered 100 mm^2^/mL. Elutes were harvested at 1, 3 and 7 days. Afterwards, the extracts were passed through 0.22 µm filters to ensure that sterile conditions were met and then stored at 4^°^C. 

The relative cytotoxicity of extracts was examined using 3-(4,5-dimethylthiazol-2-yl)-2,5-diphenyl tetrazolium bromide (MTT) assay. Fibroblast cells were placed into a 96-well plate at 1×10^4^ cells/well and incubated for 24 h to allow adhesion. Then, the cells were exposed to the 100 µL extract of the samples and incubated for another 24 h. In living cells, mitochondrial dehydrogenases reduced the MTT tetrazolium ring to form insoluble, purple formazan crystals. The formed crystals were dissolved in isopropanol (100 µL) and the absorbance of the resultant solution was quantified using an ELISA reader (Stat Fax 2100, USA) at 545 nm. The optical density of each sample was normalized according to the control group (culture medium with no sample). All assays were repeated 3 times to confirm reproducibility. Recorded values were qualitatively classified as severe (<30%), moderate (30%-60%), slight (60%-90%) and non-cytotoxic (>90%) [16].

***Sealing ability***

Thirty-six extracted human single rooted premolar teeth (with no caries, cracks, root resorption, root length shorter than 15 mm or open apices) were chosen. The teeth were decoronated with diamond discs leaving a uniform 15-mm root section. 

**Figure 1:**
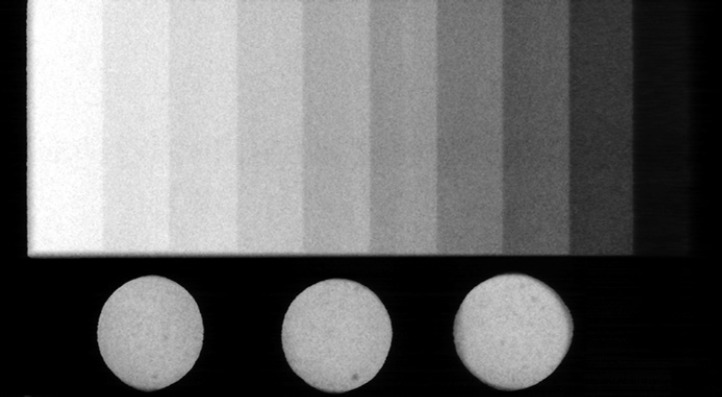
Digital radiographic image of HealApex with an aluminum step-wedge graduated from 0.5 to 5 mm Al (in 0.5-mm increments

The length of the canals was determined by passing a size 15 K-File (Dentsply Maillefer, Ballaigues, Switzerland) into the canal until its tip was visible at the apical foramen. Working length was established 1 mm short of the canal length. The coronal third of the canals was flared using #2 to #4 Gates Glidden drills. Apical portions of roots were instrumented to size 50 using step-back technique. The patency of the apical foramen was maintained with a size 10 K-file. Between the changes of the files, the canals were irrigated with 1 mL of 5.25% NaOCl solution followed by 1 mL of distilled water. 

Root canals were then dried with paper points. Standardized size 50 gutta-percha master cones were fitted to the working length with a tug back. The specimens were divided into two groups, each including 15 teeth, and filled with the sealers and accessory cones with laterally condensed gutta-percha. Six teeth were used for controls. In the positive control group, root canals were filled with a gutta-percha cone only. In the negative control group, the canals were prepared as in AH-26 group. Access cavities were sealed with dental composite (Filtek ^TM^ Z250 Universal Restorative, USA). Then, each tooth was covered with a wet gauze soaked in phosphate buffer solution. These samples were placed in an incubator at 37^°^C for 7 days to allow sealers to set. Before measurement of the leakage, the samples were coated with two layers of nail varnish except for a 2-mm area around the apical foramen. 

In this study, apical microleakage was measured using a fluid transport model described by Wu *et al.* [17]. The pressure of 0.2 atm forced the water to pass through any voids along the root filling, displacing the air bubble in the 0.1 mL capillary glass tube connected to the root section. Measurements were made at 2-min intervals in a period of 8 min. The fluid flow rate was measured on days 1, 7, and 30 and expressed in µL/min^-1^/cm H_2_O^-1^.

***Statistical analysis***

Results were statistically analyzed by t-test and analysis of variance (ANOVA), using the SPSS software version 18. The significance level of 0.05 was chosen. 

**Figure 2:**
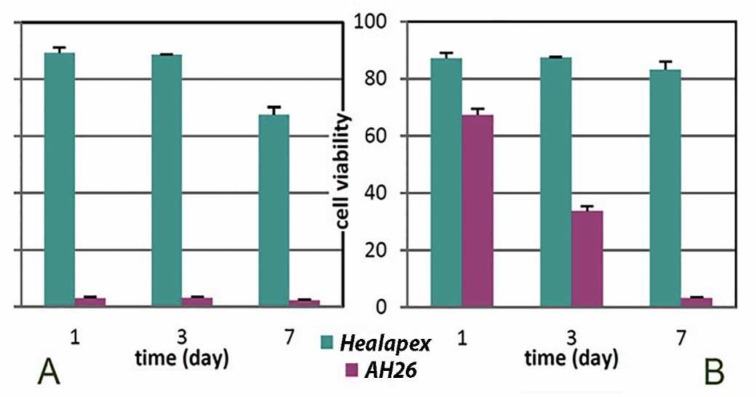
Viability of L929 cells exposed to the extracts of (A) the fresh and (B) the set sealers at different time intervals. The culture medium with no sample was considered as control

## Results

The mean values and standard deviations of physical properties (setting time, working time, film thickness, flow and radiopacity) of the tested sealers as well as standard ISO 6876 (2012) specifications are presented in Table 1. Physical properties of both sealers conformed to the ISO standard of root canal sealing materials. There were significant differences between the results of physical properties of AH-26 and HealApex (*P*<0.05). AH-26 exhibited significantly higher flow and radiopacity, and lower film thickness than that of HealApex; whereas the setting time of HealApex was lower (*P*<0.05). Figure 1 shows the digital radiographic image of HealApex, which confirms the results in Table 1. Dentin presented a radiopacity value equivalent to 0.97±0.07 mm Al.

Cell viabilities of extracts derived from fresh and set AH-26 and HealApex during 7 days are presented in Figure 2. When cells encountered the elutes of freshly sealers, AH-26 was strongly cytotoxic. There was no difference between the results of different days (*P*>0.05). Contrary to fresh AH-26, extracts from the fresh HealApex exhibited slight cytotoxicity. For this sealer, significant difference in cell viabilities was found between day 7 and other days (*P*<0.05) (Figure 2A). In set sealers, extracts of HealApex decreased cell viability slightly in comparison with the control sample during 7 days. However, set AH-26 showed moderate toxicity (Figure 2B). AH-26 was more cytotoxic than HealApex during all periods (*P*<0.05). 

The results of quantitative evaluation of sealers’ apical microleakage are illustrated in Table 2. There was no significant difference between the 2 sealers on days 1 and 7. However after 30 days, teeth filled with AH-26 sealer showed higher microleakage than those filled with HealApex (*P*<0.05). HealApex also demonstrated less microleakage on other days, although this was not significant. It was observed that AH-26 sealer had a significant increase of microleakage after the first week (*P*<0.05). During the experiment, there was no statically significant difference in the results of leakage for HealApex. The positive controls demonstrated high levels of microleakage at all-time intervals, whereas negative controls did not leak in the same time period.

## Discussion

The results of this study demonstrated that HealApex meets the requirements of ISO 6876 specifications. This new premixed calcium silicate-based sealer is biocompatible. It also has good sealing ability comparable to AH-26. Long term sealing of this biomaterial was better than the epoxy resin sealer.

Root canal sealers are in contact with vital tissue *via* the main path of communication, apical foramen. Due to the risk of overfilling during endodontic procedures, not only acceptable degree of biocompatibility is essential, but also the ability of hard tissue induction *i.e.* cementogenesis is expected [18, 19]. Extrusion of these materials may cause unfavorable consequences, such as inflammation and intense neurotoxicity [20]. Premixed calcium silicate-based sealers have been introduced to the profession for their biological benefits, principally their bioactivity potential [12]. This study compared the cytotoxicity of HealApex and AH-26 sealers *via* the exposure of extracts of materials to L929 fibroblast cells. Utilization of established cell lines, such as human gingival fibroblast or mouse fibroblasts, enables good reproducibility for *in vitro* cytotoxicity assessments [21]. This cell line can easily be prepared and cultured. In the present study, MTT assay was used for evaluating the cytotoxicity of materials. The advantages of this method its reliability, reproducibility, simplicity, speed and precision [22]. 

Premixed calcium silicate-based sealers need body tissue fluids during their setting process. Exchanging of water-free carrier with body tissue moisture leads to a hydration reaction of active ingredients and generation of calcium silicate hydrate and calcium hydroxide; all of which have the potential for bioactivity. Obtained results revealed that both freshly mixed and set HealApex have acceptable levels of biocompatibility. The fresh state of HealApex exhibited very good cell viability, which decreased slightly on day 7. It seems that such minor decrease is the consequence of gradual release of a calcium hydroxide byproduct, which increases the local pH and kills adjacent cells. The increased pH also provides the sealer with additional antibacterial characteristics. Such a property may partly have a conflict with tissue compatibility.

Our results also demonstrated that fresh AH-26 has a severe cytotoxic effect. This phenomenon could be explained by the formation of formaldehyde *via* hydrolysis of the hexamethylene tetramine to ammonia and formaldehyde. Release of free monomers during conversion process may be another reason for cytotoxicity [23]. This material has antibacterial effects, which may conflict with its biocompatibility [24]. 

Some case reports describe paresthesia and allergic reactions to AH-26 shortly after the application of the material [25, 26]. In this study, the set AH-26 exhibits moderate toxicity on L929 cells. Researchers reported strong to moderate toxicity for the set AH-26 sealer [27]. The discrepancy between the results of various studies may be due to using different time intervals between mixing and exposure of the material to cells. Other important factors are employing different cell lines, incubation time, frequency of changing the medium, methods of presenting the material to cells (direct contact or extract of material) and concentration of the material in the media [28]. 

The majority of endodontic failures caused by the imperfect apical/coronal seal of the root canal system [29, 30]. This study evaluated the sealing property of the new sealer for 30 days with the fluid transport model described by Wu *et al.* [17]. The major advantage of this model was the ability to provide a quantitative measurement of microleakage at intervals over extended periods without destroying the root specimens. In addition, this method is more sensitive than passive dye penetration for the detection of full-length voids along root canals and is highly reproducible [31]. To reduce variation, the length of all roots investigated within this study were kept identical; the diameter and anatomy of the foramen were also controlled. 

The results of the microleakage test showed the importance of a sealer in the quality of the gained seal. Samples in the positive control group (no sealer) demonstrated the highest values of microleakage. In addition, AH-26 sealer showed increased microleakage after 30 days. Researchers have previously reported an early expansion for this sealer as it sets. However, after 30 days, they observed some shrinkage, which can fracture bonding to the dentin and lead to the formation of gaps between the sealer and root canal walls [32]. This may be the reason for the highermicroleakage values after 30 days in the present study. These results are in agreement with previous studies, which have shown increased microleakage of AH-26 over time [33].

**Table 1:** Physical properties of HealApex and AH-26 sealers and compliance to ISO 6876

	**Working time (h)**	**Setting time (h)**	**Film thickness (µm)**	**Flow (mm)**	**Radiopacity (mm Al)**
**HealApex**	>4	2.2 (0.1)	37.37 (6.58)	21.51 (0.63)	3.48 (0.14)
**AH-26 sealer**	>5	9.4 (0.2)	28.8 (3.33)	27.03 (0.38)	6.37 (0.23)
***P*** **-value**	-	<0.001	0.0167	<0.001	<0.001
**ISO 6876/2012**	-	-	<50	>17	>3

**Table 2:** Leakage of tested sealers on different days (µL.min ^-1^. cm H_2_O^-1^×10^-3^)

	**1** ^th^ ** day**	**7** ^th^ ** day**	**30** ^th^ ** day**
**HealApex**	0.47 (0.37)^a^	0.47 (0.27)^a^	0.46 (0.28)^a^
**AH-26 sealer**	0.71 (0. 51)^a^	0.73 (0.46)^a^	1.31 (0.53)^b^

*T* *he means having same superscripts at the same column are not statistically different at P>0.05*

Long term sealing ability of a sealer is clinically important [34] In previous studies, it was shown that the sealing ability of calcium silicate-based sealers were comparable to epoxy resin-based sealers [35]. The results of the present study demonstrated better long term sealing ability for HealApex compared with epoxy resin.

In addition, the leakage of the new sealer with higher film thickness was less than that of AH-26 after 30 days. These results indicate that lower film thickness does not necessarily result in better sealing. Other properties are important too. Calcium silicate-based sealers do not shrink during setting and show a slight expansion through continuous hydration after initial setting of the material and further crystalline maturation [8]. The hydration reaction of these materials results in the production of calcium hydroxide. It is well established that this product can stimulate biological closure of the apical region through hard tissue formation [11]. These sealers have also demonstrated better adaption to gutta-percha than resin-based sealers [35]. However, it is still important that the film thickness of a sealer should comply with the standard specifications, due to its effects on handling and possible interference with the appropriate placement of gutta-percha cones into the root canals in condensation techniques.

Rheological properties of a sealer are important in inserting the core material and display its potential to penetrate into irregularities and accessory canals [36]. However, it has been reported that there is no correlation between the penetration of sealer into dentinal tubules and microleakage results [37].

Higher film thickness and lower flow of the new sealer in comparison with AH-26 may be due to its different composition and particle size. Other factors affecting these properties are solid concentration, particle size distribution, rate of shear, temperature and mixing time [4, 38]. In the present study, the film thickness and flow values of AH-26 sealer were 28.8 ± 3.33 µm and 27.03 ± 0.38 mm, respectively. These results agree with previous studies which showed the film thickness of AH-26 sealer to range from 26 to 39 µm [39]. Flow of this sealer has also been reported to vary from 25 to 30 mm [40, 41].

Setting time of a sealer must be long enough to allow placement and adjustment of the root filling material. However, a prolonged and lengthy setting time is a disadvantage, since washout and microleakage of the unset sealer may occur. A sealer must also have ample working time to allow mixing and manipulation of the material [42]. 

The contrast created by radiography makes the material distinguishable from surrounding anatomic structures and thus, allows clinicians to evaluate the obturation quality. The ISO 6876 standard expresses that root canal sealers should have a radiopacity value not less than 3 mm Al. In the present study, tested sealers showed higher radiopacity than the standard specification. The radiopacity value of AH-26 sealer was higher than that of HealApex. This difference may be the result of the presence of different radiopacifiers in each sealer. AH-26 radiopacity is provided by bismuth oxide while zirconium oxide acts as a radiopacifier in HealApex. In this research, we used digital radiography instead of conventional x-ray imaging. The determined radiopacity of AH-26 was 6.37. This result is in accordance with another study which reported value of 6.29 by digitized film [43].

Similar to calcium-silicate based materials, HealApex is likely to have some drawbacks [44]; therefore, further researches are necessary for improving the characteristics as well as the investigating other aspects of biocompatibility and clinical performance of this novel endodontic sealer.

## Conclusion

This *in vitro *study is the first investigation to evaluate the properties of a newly developed sealer (HealApex). The sealer exhibited acceptable physical properties and biocompatibility. In addition, sealing ability of HealApex was similar to AH-26. 

## References

[1] Lee K-W, Williams MC, Camps JJ, Pashley DH (2002). Adhesion of endodontic sealers to dentin and gutta-percha. J Endod.

[2] Grossman LLI, Oliet S, Del Rio CE (1988). Endodontic practice.

[3] Jafari F, Jafari S, Etesamnia P (2017). Genotoxicity, Bioactivity and Clinical Properties of Calcium Silicate Based Sealers: A Literature Review. Iran Endod J.

[4] Ørstavik D (2005). Materials used for root canal obturation: technical, biological and clinical testing. Endod Topics.

[5] Asgary S, Eghbal MJ, Bagheban AA (2017). Long-term outcomes of pulpotomy in permanent teeth with irreversible pulpitis: A multi-center randomized controlled trial. Am J Dent.

[6] Fallahinejad Ghajari M, Asgharian Jeddi T, Iri S, Asgary S (2013). Treatment outcomes of primary molars direct pulp capping after 20 months: a randomized controlled trial. Iran Endod J.

[7] Kollmuss M, Preis CE, Kist S, Hickel R, Huth KC (2017). Differences in physical characteristics and sealing ability of three tricalcium silicate-based cements used as root-end-filling materials. Am J Dent.

[8] Ken Koch DB (2009). Bioceramic technology – thegame changer in endodontics.

[9] Zhang H, Shen Y, Ruse ND, Haapasalo M (2009). Antibacterial activity of endodontic sealers by modified direct contact test against Enterococcus faecalis. J Endod.

[10] Gandolfi M, Spagnuolo G, Siboni F, Procino A, Rivieccio V, Pelliccioni G, Prati C, Rengo S (2015). Calcium silicate/calcium phosphate biphasic cements for vital pulp therapy: chemical-physical properties and human pulp cells response. Clin Oral Investig.

[11] Sarkar N, Caicedo R, Ritwik P, Moiseyeva R, Kawashima I (2005). Physicochemical basis of the biologic properties of mineral trioxide aggregate. J Endod.

[12] Almeida LHS, Moraes RR, Morgental RD, Pappen FG (2017). Are Premixed Calcium Silicate–based Endodontic Sealers Comparable to Conventional Materials? A Systematic Review of In Vitro Studies. J Endod.

[13] Spangberg L, Langeland K (1973). Biologic effects of dental materials: 1 Toxicity of root canal filling materials on HeLa cells in vitro. Oral Surg Oral Med Oral Pathol.

[14] Parirokh M, Forghani FR, Paseban H, Asgary S, Askarifard S, Esmaeeli Mahani S (2015). Cytotoxicity of two resin-based sealers and a fluoride varnish on human gingival fibroblasts. Iran Endod J.

[15] Duarte MAH, D'arc de Oliveira El G, Vivan RR, Tanomaru JMG, Tanomaru Filho M, de Moraes IG (2009). Radiopacity of Portland cement associated with different radiopacifying agents. J Endod.

[16] ISOstandards (2009). 10993-5 Biological evaluation of medical devices. Tests for cytotoxicity: in vitro methods.

[17] WU MK, Gee AD, Wesselink P, Moorer W (1993). Fluid transport and bacterial penetration along root canal fillings. Int Endod J.

[18] Schmalz G, Arenholt-Bindslev D (2009). Biocompatibility of dental materials.

[19] Nosrat A, Asgary S, Eghbal MJ, Ghoddusi J, Bayat-Movahed S (2011). Calcium-enriched mixture cement as artificial apical barrier: A case series. J Conserv Dent.

[20] Kılkış BT, Er K, Taşdemir T, Yildirim M, Taskesen F, Tümkaya L, Kalkan Y, Serper A (2015). Neurotoxicity of various root canal sealers on rat sciatic nerve: an electrophysiologic and histopathologic study. Clin Oral Investig.

[21] Asgary S, Moosavi SH, Yadegari Z, Shahriari S (2012). Cytotoxic effect of MTA and CEM cement in human gingival fibroblast cells Scanning electronic microscope evaluation. N Y State Dent J.

[22] Huang FM, Tai KW, Chou MY, Chang YC (2002). Cytotoxicity of resin-, zinc oxide-eugenol-, and calcium hydroxide-based root canal sealers on human periodontal ligament cells and permanent V79 cells. Int Endod J.

[23] Goldberg M (2008). In vitro and in vivo studies on the toxicity of dental resin components: a review. Clin Oral Investig.

[24] Geurtsen W, Leyhausen G (1997). Biological aspects of root canal filling materials–histocompatibility, cytotoxicity, and mutagenicity. Clinical Oral Investigations.

[25] Spielman A, Gutman D, Laufer D (1981). Anesthesia following endodontic overfilling with AH26: report of a case. Oral Surg Oral Med Oral Pathol.

[26] Barkhordar RA, Nguyen NT (1985). Paresthesia of the mental nerve after overextension with AH26 and gutta-percha: report of case. J Am Dent Assoc.

[27] Osorio RM, Hefti A, Vertucci FJ, Shawley AL (1998). Cytotoxicity of endodontic materials. J Endod.

[28] Torabinejad M, Parirokh M (2010). Mineral trioxide aggregate: a comprehensive literature review—part II: leakage and biocompatibility investigations. J Endod.

[29] Ingle JI TJ (1985). Endodontics.

[30] Yavari HR, Samiei M, Shahi S, Aghazadeh M, Jafari F, Abdolrahimi M, Asgary S (2012). Microleakage comparison of four dental materials as intra-orifice barriers in endodontically treated teeth. Iran Endod J.

[31] WU MK, GEE A, Wesselink P (1994). Fluid transport and dye penetration along root canal fillings. Int Endod J.

[32] Wiener BH, Schilder H (1971). A comparative study of important physical properties of various root canal sealers: II Evaluation of dimensional changes. Oral Surg Oral Med Oral Pathol.

[33] Wedding JR, Brown CE, Legan JJ, Moore BK, Vail MM (2007). An in vitro comparison of microleakage between Resilon and gutta-percha with a fluid filtration model. J Endod.

[34] Kontakiotis E, WU MK, Wesselink P (1997). Effect of sealer thickness on long–term sealing ability: a 2–year follow–up study. International Endodontic Journal.

[35] Zhang W, Li Z, Peng B (2009). Assessment of a new root canal sealer's apical sealing ability. Oral Surg Oral Med Oral Pathol Oral Radiol Endod.

[36] Ørstavik D (1982). Seating of gutta-percha points: effect of sealers with varying film thickness. Journal of endodontics.

[37] Generali L, Prati C, Pirani C, Cavani F, Gatto M, Gandolfi M (2017). Double dye technique and fluid filtration test to evaluate early sealing ability of an endodontic sealer. Clin Oral Investig.

[38] He M, Wang Y, Forssberg E (2004). Slurry rheology in wet ultrafine grinding of industrial minerals: a review. Powder Technology.

[39] Wu M-K, De Gee AJ, Wesselink PR (1997). Leakage of AH26 and Ketac-Endo used with injected warm gutta-percha. J Endod.

[40] Bernardes RA, de Amorim Campelo A, Junior DSS, Pereira LO, Duarte MAH, Moraes IG, Bramante CM (2010). Evaluation of the flow rate of 3 endodontic sealers: Sealer 26, AH Plus, and MTA Obtura. Oral Surg Oral Med Oral Pathol Oral Radiol Endod.

[41] Ashraf H, Najafi F, Heidari S, Mohammadian M, Zadsirjan S (2017). Physical Properties and Chemical Characterization of Two Experimental Epoxy Resin Root Canal Sealers. Iran Endod J.

[42] Gatewood RS (2007). Endodontic materials. Dent Clin North Am.

[43] Guerreiro-Tanomaru JM, Duarte MAH, Gonçalves M, Tanomaru-Filho M (2009). Radiopacity evaluation of root canal sealers containing calcium hydroxide and MTA. Braz Oral Res.

[44] Parirokh M, Asgary S, Eghbal MJ, Kakoei S, Samiee M (2011). A comparative study of using a combination of calcium chloride and mineral trioxide aggregate as the pulp-capping agent on dogs' teeth. J Endod.

